# Resolution and Frequency Effects on UAVs Semi-Direct Visual-Inertial Odometry (SVO) for Warehouse Logistics

**DOI:** 10.3390/s22249911

**Published:** 2022-12-16

**Authors:** Simone Godio, Adrian Carrio, Giorgio Guglieri, Fabio Dovis

**Affiliations:** 1 Department of Mechanical and Aerospace Engineering (DIMEAS), Politecnico di Torino, Corso Duca degli Abruzzi, 24, 10128 Turin, Italy; 2 Dronomy, Paseo de la Castellana, 40, 28046 Madrid, Spain; 3 Department of Electronics and Telecommunications (DET), Politecnico di Torino, Corso Duca degli Abruzzi, 24, 10128 Turin, Italy

**Keywords:** visual inertial odometry, autonomous localization, aerial system, SVO, warehouse, ROS, indoor localization, resolution, frequency

## Abstract

For the commercial sector, warehouses are becoming increasingly vital. Constant efforts are in progress to increase the efficiency of these facilities while reducing costs. The inventory part of the goods is a time-consuming task that impacts the company’s revenue. This article presents an analysis of the performance of a state-of-the-art, visual-inertial odometry algorithm, SVO Pro Open, when varying the resolution and frequency of video streaming in an industrial environment. To perform efficiently this task, achieving an optimal system in terms of localization accuracy, robustness, and computational cost is necessary. Different resolutions are selected with a constant aspect ratio, and an accurate calibration for each resolution configuration is performed. A stable operating point in terms of robustness, accuracy of localization, and CPU utilization is found and the trends obtained are studied. To keep the system robust against sudden divergence, the feature loss factor extracted from optical sensors is analyzed. Innovative trends and translation errors on the order of a few tens of centimeters are achieved, allowing the system to navigate safely in the warehouse. The best result is obtained at a resolution of 636 × 600 px, where the localization errors (x, y, and z) are all under 0.25 m. In addition, the CPU (Central Processing Unit) usage of the onboard computer is kept below 60%, remaining usable for other relevant onboard processing tasks.

## 1. Introduction

As the market rises due to the strong demand and diversification of products, warehouse logistics play an increasingly important role in the management of goods and delivery times. The latest are relevant parameters for the customer in which there is strong competition [1,2]. As a consequence of the pandemic period also, the e-commerce sector is growing fast and the only way to supply the chain is to go towards smart and autonomous warehouses [3]. For these reasons, robotic applications are growing in daily operations inside warehouses [4,5,6]. Several companies are already using this technology to speed up their operations while many others are on their way to incorporating it. However, to increase the autonomy level in these GPS-denied environments, it is first required to solve the problem of localizing the robot with adequate accuracy. If an inaccurate or unstable localization system is adopted, the probability of failing the task is high and even worse, the safety of humans can be compromised, especially if UAVs (Unmanned Aerial Vehicles), commonly known as drones, are employed. Therefore, an efficient localization system at its base is necessary [7]. Some of the state-of-the-art techniques for localization are presented in the following subsection. This work aims to analyze a low-cost and lightweight system that estimates the relative 3D position of a UAV inside a warehouse with respect to a known starting pose. Given the optical sensor and the available computational power, the goal is to find a stable operating point in terms of robustness, accuracy of localization, and CPU utilization, and to study the trends obtained. The localization problem itself has already been addressed with various approaches. Many of these need external access points to triangulate the robot’s position [8]. Others use heavy and expensive hardware such as LIDAR [9], not compatible with the small size required for UAVs to safely navigate along warehouse corridors. Instead, the system analyzed is independent of external aids and uses only the visual-inertial sensors onboard. In addition, a warehouse-specific training dataset is not needed as in [10]. The configuration presented has the advantage of being a low-cost and lightweight system and is easily adaptable to most warehouses and platforms. However, an accurate calibration phase is crucial to obtain an accurate and robust localization using only the onboard visual-inertial sensors [11,12,13,14,15]. To not overload the CPU, it is necessary to find a trade-off between the optical resolution adopted and the image acquisition frequency. This trade-off point is one of the main goals of the work. In addition, a detailed study of localization errors along the three dimensions is presented, not available in similar works such as [16]. Through the appropriate parameters, to minimize the risk of sudden divergence in the localization, the robustness of the system is also monitored. Lately, the use of this technology is growing fast in aerospace robotics, for terrestrial and non-terrestrial applications. For these reasons, the analyzed system suits both aerial and ground platforms. In this case, an aerial system with a quadcopter configuration is employed. UAVs unlike ground robots allow inventorying the shelves at every level, saving more time and reducing the risks that an operator would run by using traditional methods. Furthermore, thanks to this system, the drone can be programmed to be completely autonomous, and therefore, there is potentially no need for human pilot assistance.

The onboard computer employed is lightweight, inexpensive, and commercially available: NVIDIA Jetson Nano board. The optical sensor used is a fisheye stereo camera with an integrated IMU. The project in question uses a programming framework that is widespread in robotics, ROS (Robot Operating System). This system works as an operating system that connects different processes and commonly used applications. ROS organizes its content into packages containing executable files called *nodes*, programmed in C/C++, Python, and LISP. For the estimation of the noise parameters of the IMU (Inertial Measurement Unit), the ROS package IMU_utils is adopted. For the calibration of the optical system, the ROS package camera_calibration is used, while for the calibration of the visual-inertial system, the ROS package kalibr is adopted. Similarly, the localization algorithm implemented in this project is SVO Pro Open, described in [17,18], always compatible with ROS. The latter package is chosen for localization after several tests among the various open-source packages available, cited in the next section. This is one of the few packages able to run in real-time on the selected onboard computer and provide satisfactory performance. The ROS version installed is Melodic Morenia, compatible with Ubuntu 18, on both the Jetson Nano and the laptop used for the calibration phase. The experimental results presented are derived from data recorded during tests in an actual warehouse, and fully belong to a real industrial scenario.

The paper is organized as follows. The following subsection presents an overview of the state-of-the-art of visual-inertial odometry algorithms. Section 2 shows the methodology of data collection and hardware setup. This section presents the sensors adopted and the calibration process of the cameras and the visual-inertial system. Section 3 describes the obtained results and discussions. Conclusions and further developments are described in Section 4.

### 1.1. Related Work

As anticipated, one of the limits in mobile robotic applications is the uncertainty of vehicle localization. To overcome this problem, external aids such as GNSS (Global Navigation Satellite System), Motion Capture Cameras [19], Total Stations [20], or similar can be adopted. In addition, Ultra-Wideband technologies are recently taking over as a cheaper source to localize the vehicle, as described in [21]. However, these systems can rarely be employed in critical (GPS-denied) and unknown areas without additional equipment. Therefore, many of the robotic applications are quite limited in these scenarios.

For these reasons, several open-source algorithms (ORB-SLAM, SVO, VINS, Okvis, Rovio, and several others) grew up recently to perform Visual-Inertial Odometry for complete autonomous applications [22,23,24,25]. This particular technique performs a sensor fusion between optical sensors, such as monocular or stereoscopic cameras, and inertial sensors to estimate the traveled trajectory from the initial position, as explained in [26]. The process flow can be divided into (1) feature extraction from the current frame of the video stream, (2) search of the extracted features in the current frame among those of the previous one, (3) filtering features matched, (4) triangulation of the pose, and (5) fusion with the inertial data to scale the processed trajectory and refine the motion estimation. Specifically, there are relevant feature extraction, filtering techniques, and integration methods with inertial sensors, described in [17,18,22,23,24,25].

The commercial sector responded promptly by launching products such as the Intel RealSense T265 and the ZED series (ZED mini, ZED, and ZED2), ready-to-use sensors that provide directly the result of the Visual-Inertial Odometry to the user. Moreover, event cameras are also gaining ground. These are already powerful sensors for this application, even if still in a prototype state [27].

#### 1.1.1. Visual-Inertial Odometry

This term groups together those techniques consisting of combining the data coming from one or more inertial sensors with one or more RGB or depth cameras. Recently, hybrid techniques such as SVO (Semi-direct Visual Odometry) emerged also [17]. In this case, pixels are extracted with a feature-based methodology, but the variation between frames in light intensity of pixels selected for triangulation is evaluated to estimate the camera motion.

**Figure 1:**
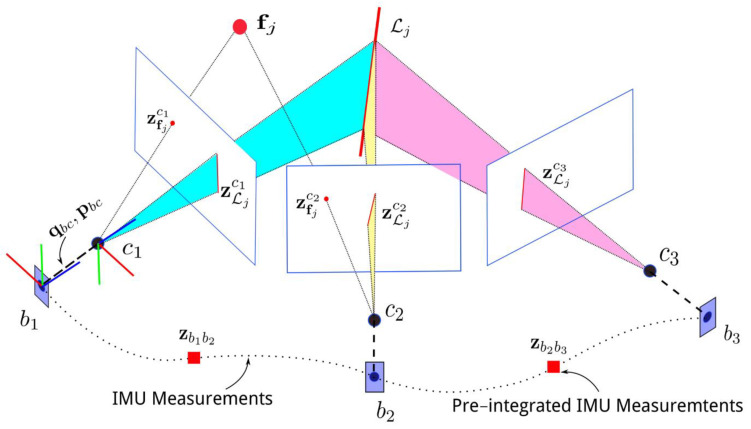
Visual-Inertial Odometry feature matching principle and IMU (Inertial Measurement Unit) measurements during motion [28].

**Figure 2:**
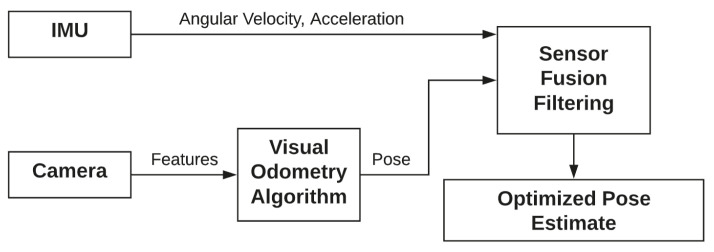
Loosely coupled sensor fusion approach.

**Figure 3:**
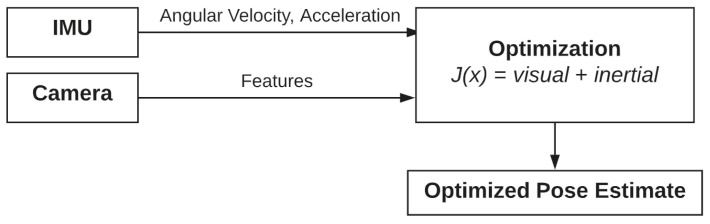
Tightly coupled sensor fusion approach.

The triangulation process and motion reconstruction are summarized in Figure 1, where the feature $f_{j}$ is recognized by two different consecutive images. It is possible to reconstruct the epipolar plane joining the two centers $c_{1}$, $c_{2}$, and $f_{j}$. Detailed equations can be found in [28]. Inertial data fusion techniques are divided into two main categories, as described in [29]:

(i) **Loosely coupled**: the visual and inertial systems are independent entities. In this case, the fusion is applied through Unscented Kalman filters or Extended Kalman Filters. Although not extremely accurate, this approach favors real-time performance. It also makes easier the integration of information coming from other sensors. The logic is represented in Figure 2.
(ii) **Tightly coupled**: this approach combines visual and inertial parameters in a single optimization problem. This approach involves the data from cameras and the IMU as described in Equation (1). It results more computationally demanding than the loosely coupled approach. As described in [30], the cost function optimization can be written as in Equation (1):
  (1) $$\begin{array}{cc} J(x) & =\underset{visual}{\underset{︸}{\sum_{i=1}^{I}\sum_{k=1}^{K}\sum_{j\in J(i,k)}e_{r}^{i,j,kT}W_{r}^{i,j,k}e_{r}^{i,j,k}}}+\\ \; & +\underset{inertial}{\underset{︸}{\sum_{k=1}^{K-1}e_{s}^{kT}W_{s}^{k}e_{s}^{k}}}, \end{array}$$
  where $e_{r}$ are the weighted reprojection errors of the camera, and $e_{s}$ are the weighted temporal errors of the IMU. Instead, *i* represents the camera index, *k* is the frame index, and *j* is the image feature index. The approach is shown in Figure 3.

In various visual-inertial odometry algorithms, some further steps and refinements are also performed. In particular, as explained in [31], loop closure, relocalization, feature retrieval, and bundle adjustment techniques are often adopted. These enhancements improve the precision of the pose estimation thanks to a global pose graph optimization using previously memorized features for relocation and to adjust the pose of current features.

#### 1.1.2. Semi-Direct Visual Odometry for Multi-Camera Systems

In particular, we are considering a system *M* with c∈C cameras. By defining $T_{CB}$ as the extrinsic calibration matrix, we can estimate the previous position of the body, $T_{BB-1}$. The process is based on a minimization of the pixel intensity residual $r_{I_{i}}c$ of the subsequent frames’ corresponding pixels. Through a projection of a known point on the scene plane $\rho_{i}\;\;\dot{=}\;{\;}_{B-1}\rho_{i}$, it is possible to identify these corresponding pixels. The projection is performed into the *C* camera’s frames in the *k* and k−1 pose, expressed respectively as $I_{k}^{C}$ and $I_{k-1}^{C}$. The residual errors’ intensity is accumulated into small patches *P* centered into the 3D point projection. The variable Δu is adopted to accumulate the intensities over each patch. The final objective of the algorithm is to find the translation and rotation of the camera, $T_{kk-1}\dot{=}\left(R,p\right)$ that minimizes the sum of the squared errors:

(2) $$\begin{array}{c} \begin{array}{cc} \left(R^{*},p^{*}\right) & =arg\;min\;C(R,p)\\ C(R^{*},p^{*}) & =\sum_{c\in C}\sum_{i=1}^{N}\sum_{\Delta u\in P}\frac{1}{2}∥r_{I_{i,\Delta u}^{c}}{∥}_{\sum_{I}}^{2}+\frac{1}{2}{∥r_{R}∥}_{\sum_{R}}^{2}+\\ & +\frac{1}{2}{∥r_{p}∥}_{\sum_{p}}^{2} \end{array} \end{array}$$

with *N* representing the number of 3-D visible points. The image intensity and prior residuals can be defined as:

(3) $$\begin{array}{cc} r_{I_{i,\Delta u}^{c}} & \dot{=}I_{k}^{C}\left(\pi \left(T_{CB}\left(R\rho_{i}+p\right)\right)+\Delta u\right)-I_{k-1}^{C}\left(\pi \left(T_{CB}\rho_{i}\right)+\Delta u\right)\\ r_{r} & \dot{=}log{\left({\tilde{R}}^{T}R\right)}^{V}\\ r_{p} & \dot{=}p-\tilde{p}. \end{array}$$

The cost function can be written as:

(4) $$C\left(R,p\right)=r{\left(R,p\right)}^{T}\sigma^{-1}r\left(R,p\right)$$

with σ representing the measurement covariance diagonal matrix. The optimization process is then solved through a Gauss–Newton logic, [32] since residuals (*R*, *p*) are not linear. For these reasons, the relations defined for the perturbations can be written in Equation (5):

(5) $$R\leftarrow R\;exp(\delta \phi^{\wedge }),\;p\leftarrow p+R\delta p$$

where ${\left(.\right)}^{\wedge }$ represents a 3 × 3 skew-symmetric matrix in the $R^{3}$ domain.

## 2. Methodology

**Figure 4:**
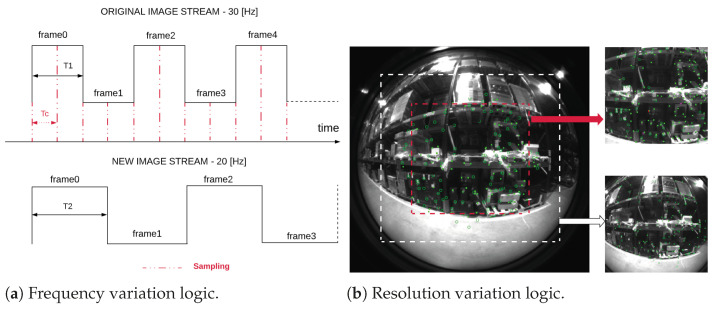
(**a**) Streaming video frequency variation example from 30 Hz to 20 Hz. (**b**) Decrease in resolution from 848 × 800 px on the center of the image.

This paper investigates the visual odometry performance under various combinations of image resolution and image acquisition frequency. Therefore, an ROS-compatible C/C++ code is developed to vary the frequency and resolution of video streaming, as illustrated in Figure 4.

Figure 4b is extrapolated from one of the recordings taken inside the warehouse during the test campaign. As shown, 3 different resolutions are tested: 848 × 800 px (original), 636 × 600 px, and 424 × 400 px. The frequencies tested can be expressed as done in Equations (6) and (7):

(6) $$\left\{\begin{array}{cc} f_{s} & =2f_{f}\\ f_{f}^{\prime } & =\frac{f_{f}}{2}+5n,\;n=0\to 3, \end{array}\right.$$

where $f_{s}$ is the sample frequency, $f_{f}$ the frame frequency, and $f_{s}^{\prime }$ is the new frame frequency tested. Instead, the resolutions tested can be expressed as:

(7) $$\left\{\begin{array}{cc} w^{\prime } & =\frac{n+1}{Fib(n)+2}w\\ h^{\prime } & =\frac{n+1}{Fib(n)+2}h,\;n=0\to 3, \end{array}\right.$$

where *w* and *h* are the original width and height, respectively, while $w^{\prime }$ and $h^{\prime }$ are the new resolution tested.

The main goal is to evaluate the impact of the resolution on the performance of the localization algorithm, as shown in the next section. The resolutions are scaled with the same center since the central zone suffers less from lens distortion. In this way, features extracted from that zone, shown in Figure 4b, suffer from a reduced error in terms of 2D to 3D projection, and the pose calculation is more accurate. In addition, this cropping excludes pixels on the edges of the image, which do not carry information on the external environment. As shown in Figure 4a, where Tc represents the sampling period, $T_{1}$ and $T_{2}$ are the original and the new streaming video frequency rate, respectively; a sampling period $Tc=2T_{1}$ is chosen to reduce the number of lost frames, but at the same time, no denser sampling is adopted so as not to further increase the computational cost. Moreover, the impact of the acquisition frequency on the performance of SVO Pro Open is also evaluated. In particular, four characteristic frequencies are selected: 30 (original), 25, 20, and 15 Hz.

### 2.1. Hardware Setup

**Figure 5:**
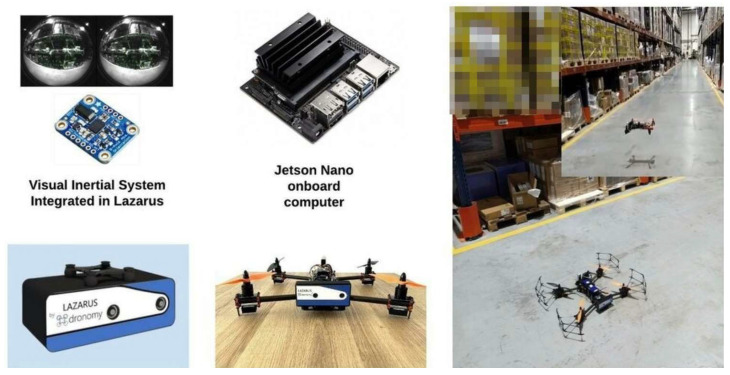
Platform employed in the warehouse: drone in a quadcopter configuration, developed by the Spanish company Dronomy.

The system proposed is accessible on multiple platforms, since a low-cost, and lightweight commercial onboard computer is adopted, as shown in Figure 5: Jetson Nano embedded system (NVIDIA Maxwell™ 128 core, ARM A57 quad-core running at 1.43 GHz, LPDDR4 4 GB 64-bit 25.6 GB/s). It is equipped with an integrated GPU (Graphics Processing Unit) that allows running simple machine learning algorithms [33]. The optical sensor used is a stereo camera with a fisheye lens, with a resolution of 848 × 800 px, hemispherical FOV (Field of View) = 163 ± 5°. The camera is part of the Lazarus device, developed by the Spanish company Dronomy to facilitate the autonomous flight of UAVs in GNSS-denied/degraded environments. The 6-axis inertial sensor is the Bosch BNI055.

### 2.2. Sensor Calibration

This section describes the calibration operations carried out to run the visual-odometry algorithm with accurate results. Firstly, the white noise and bias instability parameters for the inertial sensor adopted are extracted. Later, the two optical sensors are calibrated to extract the distortion matrix and the intrinsic parameters. Once these data are obtained, it is possible to move on to calibrate the complete visual-inertial system.

#### 2.2.1. IMU Parameter Extraction

To move on to the next stages of calibration, it is necessary to estimate the gyroscope and accelerometer noise parameters of our IMU (Inertial Measurement Unit) by analyzing the Allan Variance (Equation (8)).

(8) $$\begin{array}{c} \sigma_{y}^{2}\left(M,T,\tau \right)=\frac{1}{M-1}\{\sum_{i=0}^{M-1}[\frac{x(iT+\tau )-x(iT)}{\tau }{]}^{2}-\\ +\frac{1}{M}[\sum_{i=0}^{M-1}\frac{x\left(iT+\tau \right)-x{\left(iT\right)}^{-2}}{\tau }{]}^{2}\}, \end{array}$$

where x(t) is the clock reading measured at time *t*, *M* the number of frequency samples used in variance, *T* the time between each frequency sample, and τ is the time length of each estimation.

In particular, an accurate prediction of parameters in Table 1 allows for a more effective integration with the optical sensor data in the visual-inertial odometry. The ROS package IMU_utils extracted the results shown in Table 1 for the IMU employed, through a two-hour static acquisition.

#### 2.2.2. Camera Calibration

After extracting the IMU parameters, the stereo camera’s intrinsics and calibration parameters are obtained. This phase is extremely important for an accurate 2D to 3D reprojection of the features extracted from the images, and consequently, for an accurate estimation of the motion. The equidistant distortion model, described in [35], is adopted. This model suits well to describe sensors with high FOV and a significant distortion, as described in [36].

**Figure 6:**
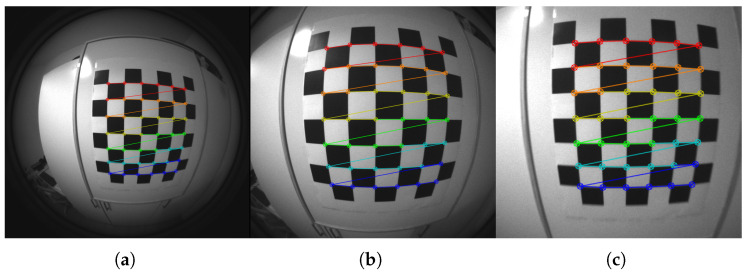
(**a**) 848 × 800 px; (**b**) 636 × 600 px; (**c**) 424 × 400 px. ROS camera-calibration process capture for each of the resolutions selected with the respective target feature extraction. The marker employed is a 7 × 6 chessboard with a square size of 5.9 cm.

It is possible to obtain accurate calibration parameters using the ROS camera-calibration package. Figure 6 shows some capture during this process with the respective feature extraction for each resolution tested.

Table 2 shows the calibration results for both lenses. As shown, the main differences as the resolution changes stay in the coordinates of the central point; on the other hand, the focal distances and the distortion parameters do not undergo significant changes as they are not only related to the resolution but to the sensor type.

#### 2.2.3. Visual-Inertial System Calibration

As the last calibration step, obtaining the transformation matrices imu-left camera and the imu-right camera is needed. For this purpose, the kalibr software is used on the same target of Figure 6. A parameter to approximate correctly during this process is the delay between the output of the inertial sensor and the optical sensor. These sensors are inevitably asynchronous as they operate at frequencies of different orders of magnitude: 200 Hz for the IMU and 15–30 Hz for the cameras.

**Figure 7:**
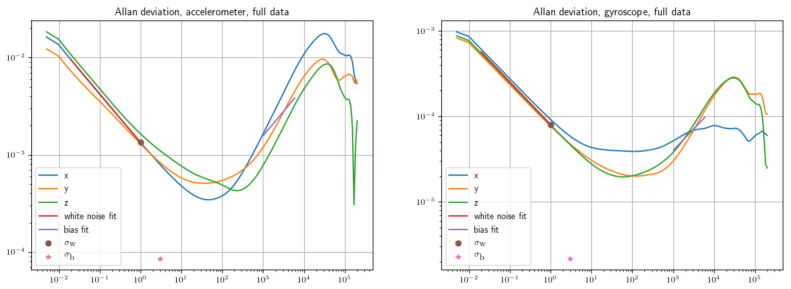
Example of modeling accelerometer and gyroscope bias by cubic B-spline.

The following assumptions are made as described in [13]: (i) IMU white noise and random walk are correctly estimated; (ii) cameras’ intrinsic and distortion parameters are known; (iii) the gravity direction can be easily guessed in the IMU values; (iv) the size of the calibration target is known so that the calibration pattern of the target can be easily reprojected in the world reference frame. In this way, it is possible to have (v) an initial guess of the calibration matrix, camera_to_imu. The time offset is initially set to zero. A first estimate of the IMU pose with respect to the two optical sensors is obtained by estimating the position of the cameras for each frame with the calibration pattern and the accelerations recorded by the IMU. Then, the IMU pose is represented by a sixth-order B-spline. The random walks are also encoded by cubic B-splines, as shown in Figure 7.

The Levenberg–Marquardt (LM) algorithm [37] is finally used to minimize the objective function to find the maximum likelihood estimate of all unknown parameters at once. This particular algorithm achieves accurate calibration parameters with reprojection errors less than 0.13 px, as demonstrated in [37]. The estimator process is illustrated in [13], and not reported to avoid unnecessary redundancies.

**Figure 8:**
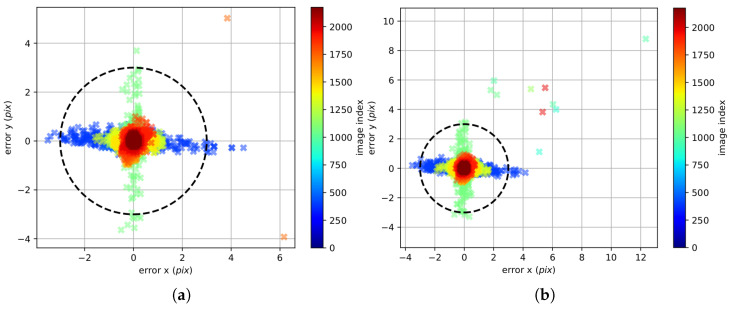
(**a**) Left cam reprojection error; (**b**) Right cam reprojection error. ROS kalibr reprojection errors after the calibration optimization process. Mean reprojection error (left cam) px: 0.1627. Mean reprojection error (right cam) px: 0.1734.

In our case, delay_imu_cam=0.098 s is obtained. Figure 8 illustrates the reprojection error obtained for the two optical sensors during the calibration phase. Usually, a value between 0.1–0.2 px is a sign of a successful calibration, as in our case where an average value of 0.1734 px is achieved.

Equations (9) and (10) show the results obtained after calibration of the visual-inertial system. In particular, the transformation matrices imu_to_left_cam and imu_to_right_cam are represented in quaternions, where the last column represents the translation vector between the two reference systems.

(9) $$\begin{array}{c} \begin{array}{cc} T\_I\_L & =\left[\begin{array}{cccc} -0.99999847 & -0.00247529 & +0.00224508 & 0.01116282\\ 0.00247665 & -0.99999675 & 0.00060109 & 0.01267902\\ 0.00224358 & 0.00060664 & 0.99999730 & -0.00601156\\ 0.0 & 0.0 & 0.0 & 1.0 \end{array}\right] \end{array} \end{array}$$

(10) $$\begin{array}{c} \begin{array}{cc} T\_I\_R & =\left[\begin{array}{cccc} -0.99999847 & -0.00171156 & -0.00036254 & -0.05166626\\ 0.00171121 & -0.99999809 & 0.00094185 & 0.01265162\\ -0.00036415 & 0.00094122 & 0.99999949 & -0.00598808\\ 0.0 & 0.0 & 0.0 & 1.0 \end{array}\right] \end{array} \end{array}$$

In addition, the parameters obtained in Equation (9) and (10) are validated by the fact that the translation values obtained are close (e<0.2 cm) to the parameters measured in the laboratory.

## 3. Results and Discussion

**Figure 9:**
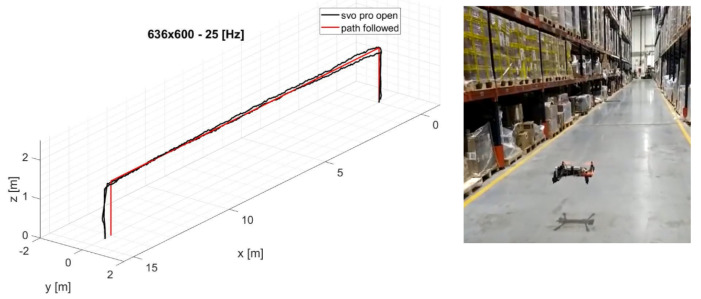
Example of a trajectory estimated inside the warehouse, with a resolution of 636 × 600 px at a sampling rate of 25 Hz.

The results shown in this section are collected on a dataset recorded within the warehouse shown in Figure 9. The trajectory performed reaches an altitude of 1.40 m after take-off, and after a translation movement along the X-axis of 14.0 m, the same path is traveled in the opposite direction to return to the starting point for landing. The goal of the aircraft in this operation is to analyze and map the parcels on the shelf at that altitude while maintaining a safe distance from it. This approach makes it possible to automate warehouse logistics procedures, reducing the time and cost of inventories.

The algorithm implemented for localization is SVO Pro Open [17]. The values analyzed in this section are translation errors, % CPU usage, and feature loss (FL). These are studied by varying the optical sensor frequency (15, 20, 25, and 30 Hz) and resolution (424 × 400, 636 × 600, and 848 × 800 px).

### 3.1. Translation Error Analysis

**Figure 10:**
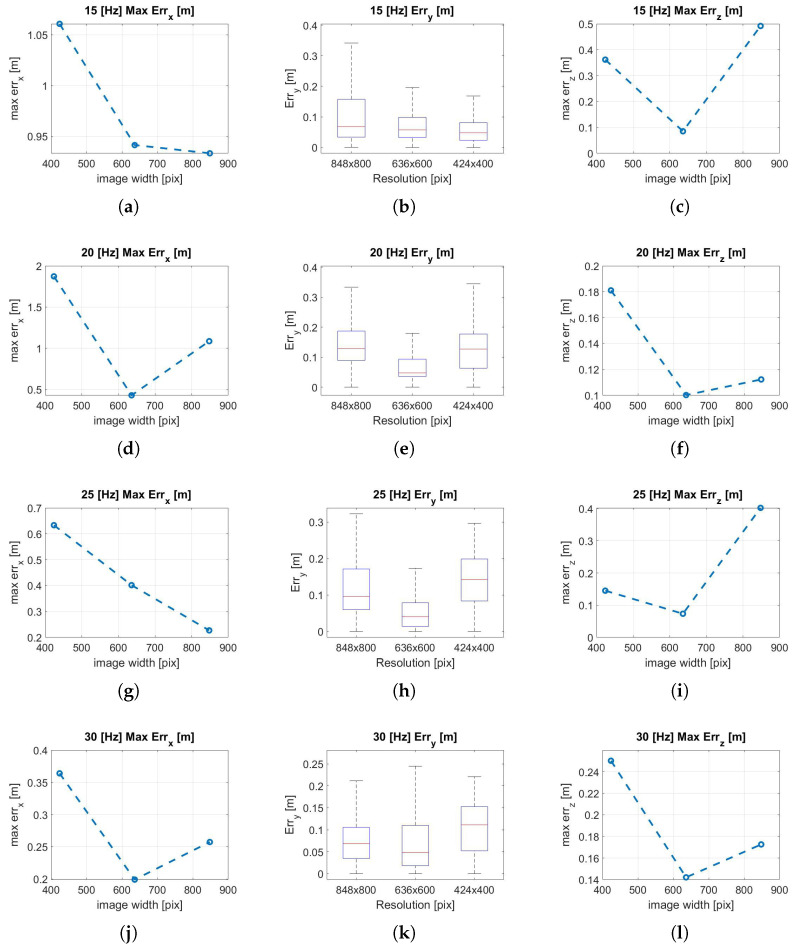
(**a**) X max error, 15 Hz; (**b**) Y error, 15 Hz; (**c**) Z max error, 15 Hz; (**d**) X max error, 20 Hz; (**e**) Y error, 20 Hz; (**f**) Z max error, 20 Hz; (**g**) X max error, 25 Hz; (**h**) Y error, 25 Hz; (**i**) Z max error, 25 Hz; (**j**) X max error, 30 Hz; (**k**) Y error, 30 Hz; (**l**) Z max error, 30 Hz. Translation error analysis along the trajectory performed by varying resolution.

**Figure 11:**
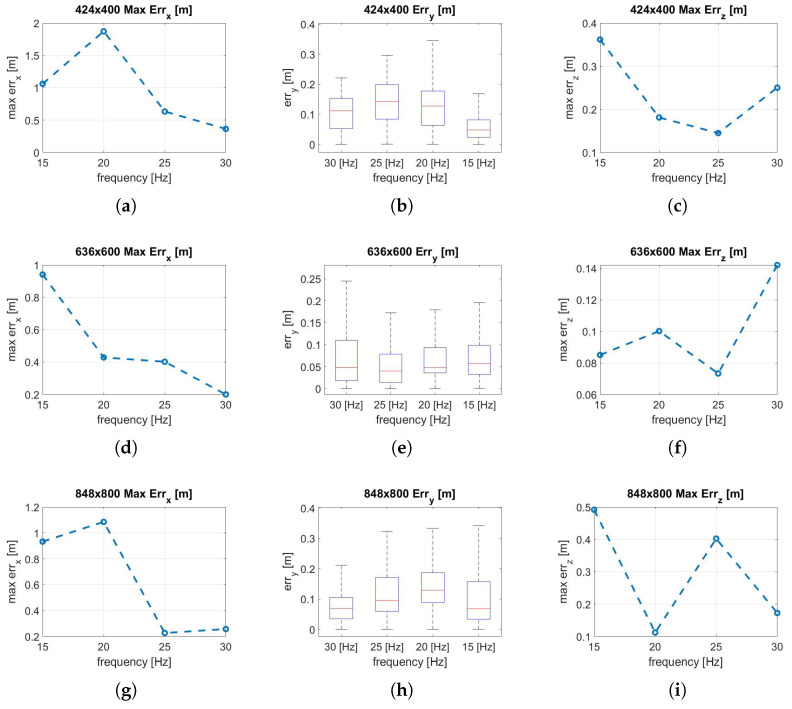
(**a**) X max err, 424 × 400; (**b**) Y err, 424 × 400; (**c**) Z max err, 424 × 400; (**d**) X max err, 636 × 600; (**e**) Y err, 636 × 600; (**f**) Z max err, 636 × 600; (**g**) X max err, 848 × 800; (**h**) Y err, 848 × 800; (**i**) Z max err, 848 × 800. Translation error analysis along the trajectory performed by varying frequency.

**Figure 12:**
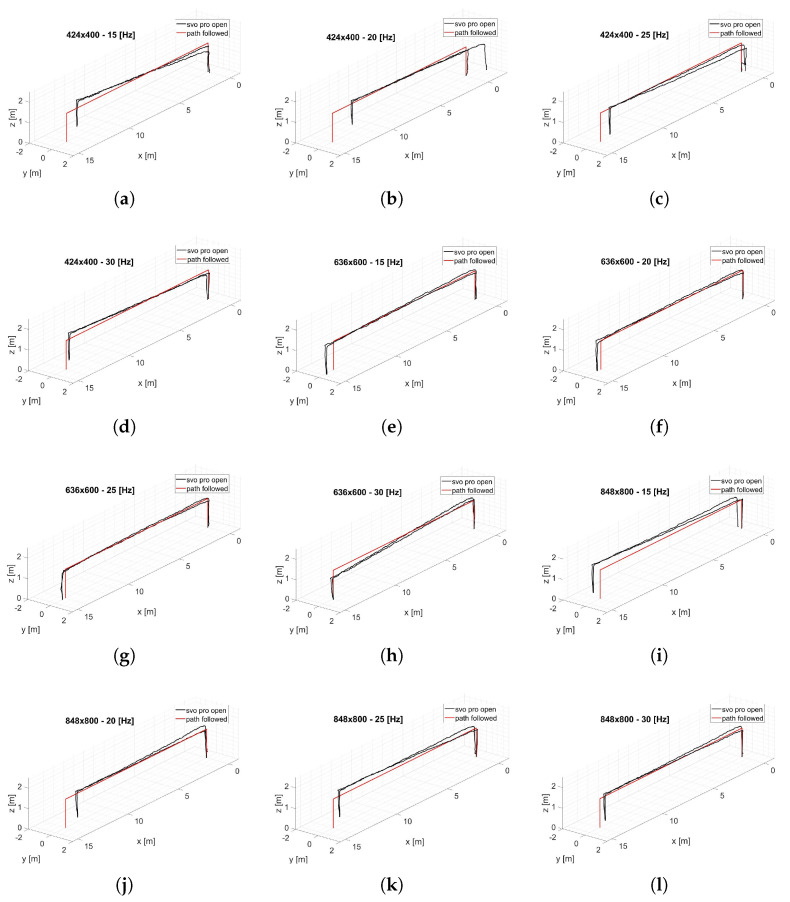
(**a**) 424 × 400, 15 Hz; (**b**) 424 × 400, 20 Hz; (**c**) 424 × 400, 25 Hz; (**d**) 424 × 400, 30 Hz; (**e**) 636 × 600, 15 Hz; (**f**) 636 × 600, 20 Hz; (**g**) 636 × 600, 25 Hz.; (**h**) 636 × 600, 30 Hz; (**i**) 848 × 800, 15 Hz; (**j**) 848 × 800, 20 Hz; (**k**) 848 × 800, 25 Hz; (**l**) 848 × 800, 30 Hz. 3D trajectory of all the conditions analyzed.

In the experiments presented in this article, the visual-inertial system is transported by hand along a predetermined linear path. To estimate the translation error along the X and Z axes, and given that there is no absolute tracking system available in this warehouse to collect the ground truth data, the maximum deviation to the values 0.0–14.0 m, and 0.0–1.40 m, is respectively considered. While along the Y-axis, the path followed is equal to y = 0 m; then, any variation from this path is considered as an error. Figure 10 shows the analysis of the effect of changing the resolution at a fixed frequency for translation errors along the X, Y, and Z axes. It is notable in almost all trends (Figure 10c–f,h–l) that an increase in resolution does not necessarily indicate an improvement in localization accuracy. Taking as reference the 30 Hz configuration that provides the best performance, the translation error trend finds a minimum point in the intermediate resolution (636 × 600 px) along all axes. In fact, increasing the resolution allows extracting more features for the same frame; however, for fisheye optical sensors, the outermost features are the ones that suffer from a higher error due to the distortion model since the features extracted are more distant from the focal point. This can cause the performance degradation in the reprojection phase as recorded at 848 × 800 px, where the minimal intensity residuals $r_{I-i}c$, described in Equation (3) optimization, lose accuracy. Figure 11 shows the effects of frequency variation on translation errors. The errors along the X and Y axes are lower at high frequency, as shown in Figure 11a,b,d,g,h. The error along the Z-axis shows random trends at higher frequencies, but at low frequencies, it can increase considerably, as shown in Figure 11c,i. In Figure 12 are represented all the trajectories extracted with the various combinations of frequency and resolution. In addition, the low-resolution trajectories (424 × 400 Hz) are the most inaccurate, as can be noted graphically in Figure 12a–c. The solution of the algorithm improves at higher frequencies.

### 3.2. Computational Cost Analysis

**Figure 13:**
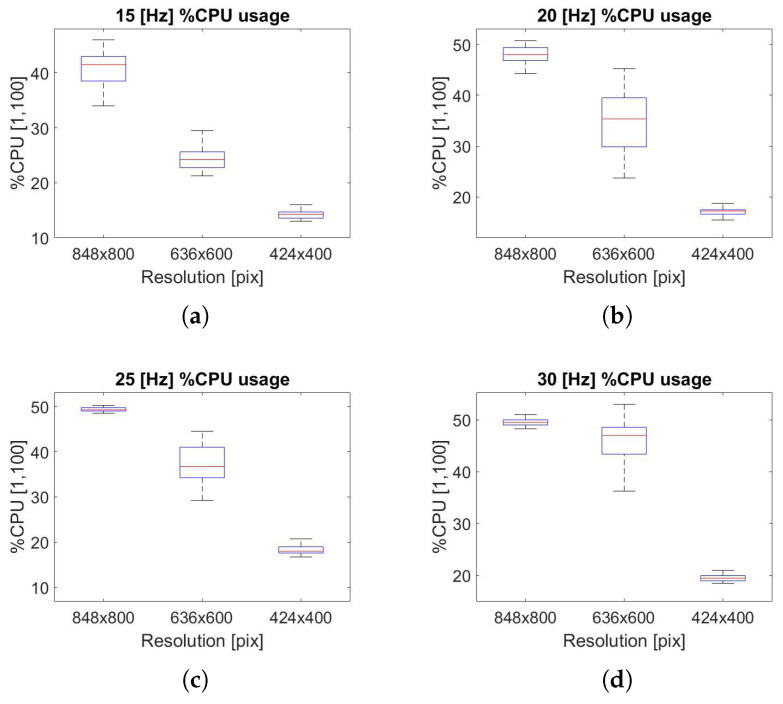
(**a**) CPU usage, 15 Hz; (**b**) CPU usage, 20 Hz; (**c**) CPU usage, 25 Hz; (**d**) CPU usage, 30 Hz. Jetson Nano board computational cost analysis along the trajectory performed by varying the resolution.

**Figure 14:**
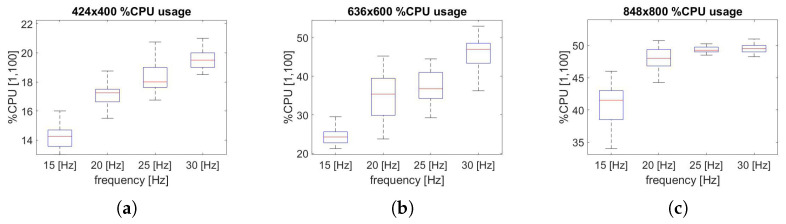
(**a**) CPU usage, 424 × 400; (**b**) CPU usage, 636 × 600; (**c**) CPU usage, 848 × 800. Jetson Nano board computational cost analysis along the trajectory performed by varying the frequency.

To perform a computational cost analysis, the impact of the SVO Pro Open process on the CPU percentage is evaluated, as shown in Figure 13 and Figure 14.

In Figure 13, it can be seen how the impact of resolution on computational cost is substantial: a reduction of up to 30% between the maximum and minimum resolution is achieved. Furthermore, as the frequency increases, the trend as the resolution changes go from linear, Figure 13a, to a function approximating an exponential trend.

A similar phenomenon is shown in Figure 14, whereas the resolution increases, the computational benefits are reduced by decreasing the frequency, changing the function from linear, Figure 14a, to a function approximating a logarithmic trend. In addition, the effect of frequency on the CPU has a major impact on the intermediate resolution (636 × 600 px), as shown in Figure 14b, while for other resolutions, it has a lower effect, Figure 14a,c. All the computational data results are collected in Table 3, Table 4 and Table 5, respectively. The theoretical explanation for this trend can be found in the definition of the cost function described in Equations (2) and (5). As *N*, the number of 3D visible point increase, more iterations are needed to elaborate the cost function, and therefore more CPU resources.

### 3.3. Feature Loss Analysis

The Feature Loss (FL) parameter is monitored to estimate the robustness of the algorithm. This parameter indicates the characteristic features that are extracted by the algorithm in one frame and not found in the next. Higher values of this parameter can lead to non-tolerable errors in the localization or, in the worst case, sudden divergences. It is important to specify that during all the tests, the maximum number of extracted features was constant.

**Figure 15:**
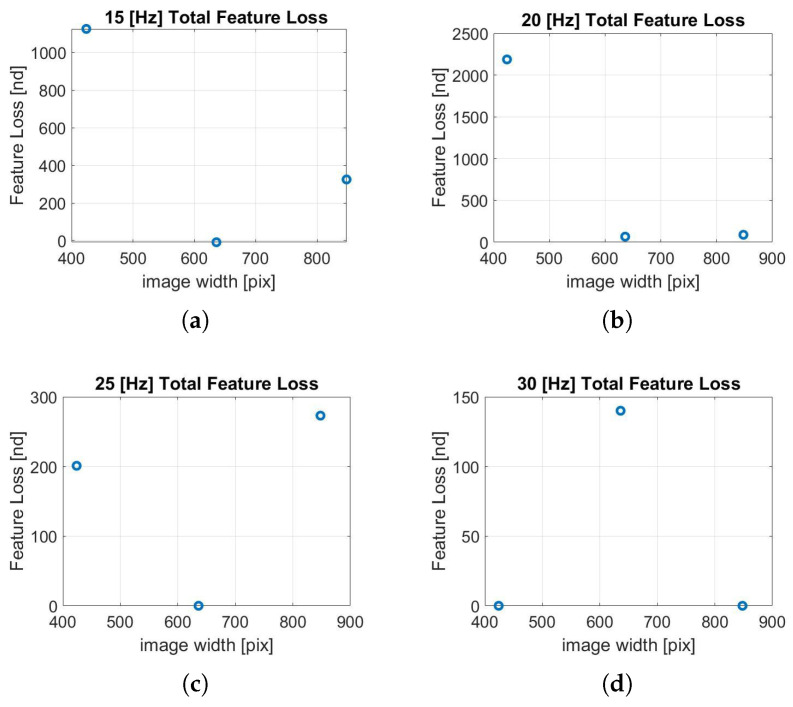
(**a**) FL, 15 Hz; (**b**) FL, 20 Hz; (**c**) FL, 25 Hz; (**d**) FL, 30 Hz. Feature loss analysis along the trajectory performed by varying resolution.

**Figure 16:**
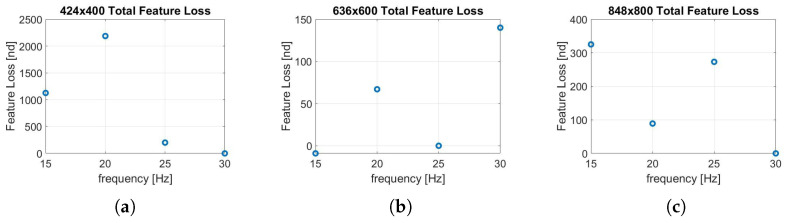
(**a**) FL, 424 × 400; (**b**) FL, 636 × 600; (**c**) FL, 848 × 800. Feature loss analysis along the trajectory performed by varying frequency.

From the several data collected, it is clear that the trend of this parameter is influenced by the calibration parameters obtained in Table 2. For this reason, no clear and unidirectional trends are highlighted in Figure 15 and Figure 16. However, it can be noted that at low frequencies, for the lowest resolution (424 × 400 px), the FL value increases by an order of magnitude, as shown in Figure 15a,b and Figure 16a. This leads to the high errors for the 424 × 400 px resolution shown earlier in Figure 12a–c. Furthermore, analyzing Figure 15, the frequency of 30 Hz shows lower values of this parameter and therefore can be considered more robust.

Instead, it can be noted from Figure 16 that the intermediate resolution (636 × 600 px) shows better performance under this aspect, showing no divergence even at low frequencies. Naturally, lowering the frequency increases the time between frames; consequently, in some phases of the test, the motion of the camera can be wider than when sampling at higher frequencies. This can compromise the Feature Matching process and therefore increase the Feature Loss (FL) parameter.

## 4. Conclusions and Further Developments

This article presents an analysis of the performance of a state-of-the-art, visual-inertial odometry algorithm, SVO Pro Open, when varying the resolution and frequency of video streaming. The algorithm is deployed on lightweight commercial hardware to demonstrate the potential use of this technology in an industrial application and to provide a valid and useful platform configuration to the scientific community.

The results obtained for three different resolutions and four acquisition frequencies are promising. In particular, it emerges that for the analyzed intermediate resolution (636 × 600 px), an optimum compromise can be obtained in terms of localization accuracy, CPU utilization, and system robustness (i.e., feature loss). The study with the variation of the frequency shows that, at high frequencies (25 and 30 Hz), better results are obtained in terms of localization. Furthermore, from the computational analysis, it emerged that a frequency of 25 Hz allows a considerable saving in computational terms compared to 30 Hz for this intermediate resolution, albeit with slightly higher translation errors. Therefore, the user can find the appropriate trade-off, depending on the computational capabilities available. In addition to promising punctual results, one of the innovative aspects of this work is that mathematical trends are highlighted and discussed in CPU usage as the frequency and resolution of the system change. This approach opens up several possibilities for CPU savings and localization accuracy improvements without changing the sensor itself since this work demonstrates that localization quality does not necessarily improve by increasing the resolution.

As a future work, it would be interesting to estimate the trends as the frequency resolution changes when using other lenses than fisheye, and multiple visual-inertial odometry algorithms. In addition, a collection of data and a comparison between different environments would help further understand the problem in order to optimize solutions. Finally, it would be interesting to extend the system to multiple cameras (rig of cameras) and to evaluate the effects of resolution and frequency in this configuration also.

## Figures and Tables

**Table 1:** Gyroscope and accelerometer calibration parameters. Equations are derived in [34].

Parameter	Symbol	BNI055	Unit
Gyroscope “white noise”	$\sigma_{g}$	0.0018491	rad (s $\sqrt{\mathrm{Hz}}{)}^{-1}$
Accelerometer “white noise”	$\sigma_{a}$	0.01094	m (s^2^ $\sqrt{\mathrm{Hz}}{)}^{-1}$
Gyroscope “bias instability”	$\sigma_{bg}$	$2.5482\times 10^{-5}$	rad $\sqrt{\mathrm{Hz}}{\left(s\right)}^{-1}$
Accelerometer “bias instability”	$\sigma_{ba}$	0.00058973	m $\sqrt{\mathrm{Hz}}{\left(s\right)}^{-2}$

**Table 2:** Intrinsic and distortion parameters for left (*l*) and right (*r*) fisheye cameras for the three resolutions selected. The parameters $f_{x}$ and $f_{y}$ represent the focal length along X and Y. Instead, $c_{x}$ and $c_{y}$ are the principal point coordinates along X and Y. While k1, k2, k3, k4 are the distortion parameters of the equidistant camera model [36].

Param	$848\times 800_{l}$	$636\times 600_{l}$	$424\times 400_{l}$	$848\times 800_{r}$	$636\times 600_{r}$	$424\times 400_{r}$
$f_{x}$	285.3568	285.3568	285.7695	285.5315	285.5315	285.3433
$f_{y}$	285.4461	285.4461	285.6246	285.5397	285.5397	285.1813
$c_{x}$	419.0777	310.2573	207.0993	414.3119	305.4019	202.1401
$c_{y}$	399.5762	297.1926	200.8910	396.4943	294.4193	196.9490
$k_{1}$	−0.005900	−0.005900	−0.005900	−0.006894	−0.006894	−0.006894
$k_{2}$	0.04159	0.04160	0.04160	0.04397	0.04397	0.04397
$k_{3}$	−0.03861	−0.03861	−0.03861	−0.04040	−0.04040	−0.04040
$k_{4}$	0.006450	0.006451	0.006451	0.006843	0.006843	0.006843

**Table 3:** Low-resolution CPU usage values.

Freq (Hz)	Mean (% CPU)	Max (% CPU)	Min (% CPU)
15	13.968	16	5.5
20	16.578	19.25	4
25	18.148	21.5	9
30	19.056	24.25	7.5

**Table 4:** Mean-resolution CPU usage values.

Freq (Hz)	Mean (% CPU)	Max (% CPU)	Min (% CPU)
15	24.543	30.5	21.25
20	34.993	45.25	23.75
25	37.298	44.5	29.25
30	45.31	53	30.5

**Table 5:** High-resolution CPU usage values.

Freq (Hz)	Mean (% CPU)	Max (% CPU)	Min (% CPU)
15	40.658	46	34
20	48.048	50.75	44.25
25	49.467	51	48.5
30	49.637	53	48.25

## Data Availability

Contact the authors for implementation details.

## Abbreviations

The following abbreviations are used in this manuscript:

SVO	Semi-direct Visual Odometry
GPS	Global Positioning System
UAV	Unmanned Aerial Vehicle
CPU	Central Processing Unit
ROS	Robot Operating System
SLAM	Simultaneous Localization And Mapping
RGB	Red Green Blue
IMU	Inertial Measurement Unit
GPU	Graphics Processing Unit
FOV	Field Of View
FL	Feature Loss
